# Supplementary material for: Terminalia bentzoë, a Mascarene Endemic Plant, Inhibits Human Hepatocellular Carcinoma Cells Growth In Vitro via G0/G1 Phase Cell Cycle Arrest
Source: Pharmaceuticals (Basel). 2020 Oct 12;13(10):303. doi: 10.3390/ph13100303 (PMC7650599; doi:10.3390/ph13100303)
Supplement: Supplementary file 1 [file pharmaceuticals-13-00303-s001.docx]

**Supplementary Materials**

***Terminalia bentzoë*, a Mascarene endemic plant, inhibits human hepatocellular carcinoma cells growth *in vitro* via G0/G1 phase cell cycle arrest**

**Nawraj Rummun^1,2,3^, Philippe Rondeau^4^, Emmanuel Bourdon^4^_,_ Elisabete Pires^5^, James McCullagh^5^, Timothy D.W. Claridge^5^, Theeshan Bahorun^2^, Wen-Wu Li^3*^ and Vidushi S. Neergheen^1,2*^**

^1^Department of Health Sciences, Faculty of Medicine and Health Sciences, University of Mauritius, Réduit, 80837, Republic of Mauritius. [n.rajeevr10@gmail.com](mailto:n.rajeevr10@gmail.com) (NR); v.neergheen@uom.ac.mu (VSN)

^2^Biopharmaceutical Unit Centre for Biomedical and Biomaterials Research, MSIRI Building, University of Mauritius, Réduit, 80837, Republic of Mauritius. [tbahorun@uom.ac.mu](mailto:tbahorun@uom.ac.mu) (TB)

^3^School of Pharmacy and Bioengineering, Faculty of Medicine and Health Sciences, Thornburrow Drive, Stoke on Trent, ST4 7QB, Keele University, UK. [w.li@keele.ac.uk](mailto:w.li@keele.ac.uk) (WWL)

^4^Université de La Réunion, INSERM, UMR 1188 Diabète athérothrombose Thérapies Réunion Océan Indien (DéTROI), Saint-Denis de La Réunion, France. [rophil@univ-reunion.fr](mailto:rophil@univ-reunion.fr) (PR); [emmanuel.bourdon@univ-reunion.fr](mailto:emmanuel.bourdon@univ-reunion.fr) (EB)

^5^Chemical Research Laboratory, University of Oxford, Oxford, OX1 3TA, United Kingdom. elisabete.pires@chem.ox.ac.uk (EP); james.mccullagh@chem.ox.ac.uk (JM); tim.claridge@chem.ox.ac.uk (TC)

***** Correspondences: neergheen@uom.ac.mu (VSN); w.li@keele.ac.uk (WWL)

**Experimental methods**

**GC-MS ANALYSIS**

The GC-MS analysis was carried out using the Agilent 7890A gas chromatography systems. The apparatus included an HP5-MS column coupled with Agilent MS model 5975C MSD with Triple axis detector (Agilent Technologies, US). The analysis began with the initial oven temperature set at 60°C for 2min, which increased at the rate of 10°C/min to a maximum of 300°C, and was maintained for another 4 min to yield a total run of 30 min under a constant helium pressure (10psi). Trimethylsilylimidazole (TMSI) derivatization of fractions was achieved by adding 10 µL of pyridine was added, followed by 50 µL of N, O-Bis(trimethylsilyl)trifluoroacetamide (BSTFA) to an Eppendorf tube containing 1 mg of lyophilized extract and incubated at 40°C – 50°C for 1 hour. Analysis was conducted by injecting 2 µL of the Trimethylsilylimidazole (TMSI) derivatized sample into the Agilent 7890A gas chromatography systems and the chromatogram analyzed by matching the MS spectra of the peaks with those stored in the NIST 2011 Mass Spectral Library (Agilent Technologies, USA).

**LC-MS ANALYSIS**

LC-MS analysis was achieved using a HESI II electrospray ion source on a Thermo Scientific Q-Exactive Orbitrap mass spectrometer system coupled to a Waters BEH C18 (2.1x100 mm 1.7 µm particle size) reversed phase column via a Thermo Scientific Ultimate U3000 ultra-performance liquid chromatography system. Fractions or isolated compounds were separated using a linear gradient method utilising mobile phase A (0.1% formic acid in water) and mobile phase B (acetonitrile with 0.1% formic acid) at a flow rate of 0.4 mL/min. The gradient consisted: 0 min (95% A), 1 min (95% A), 7 min (100% B), 7.01 min (95% A) and 10 min (95% A). The MS method utilised a full-MS experiment in negative ion mode with 140,000 resolutions and a scan range of 300-2500.

**NMR ANALYSIS**

^1^H NMR (500 MHz), ^13^C NMR (125 MHz), and 2D-NMR (COSY, and HSQC) spectra were obtained on a Bruker AVII 500 MHz instrument equipped with a dual ^13^C/^1^H cryoprobe. Chemical shifts were reported in *δ* (ppm) referenced to the solvents shifts (methanol-d_4_ or acetone-d_6_) and coupling constants (*J*) were measured in Hertz.

**HepG2 CELL VIABILITY PROFILES**

| Cell viability (% control)  **B** | **A** |
| --- | --- |
|  |  |
|  | **C** |
|  | *T. bentzoë* crude/ fractions cconcentration (µg/mL) |

**Figure S1: Cell viability profile of HepG2 cells treated with *T. bentzoë* extract and fractions.** HepG2 cells were incubated with varying concentrations of test extracts for 48 hours and cell viability was determined using MTT assay. For each concentration, the percentage of cell viability was calculated relative to the negative control (cells treated with 0.125 % DMSO).

**HepG2 FLOW CYTOMETRIC PROFILES.**

| 2.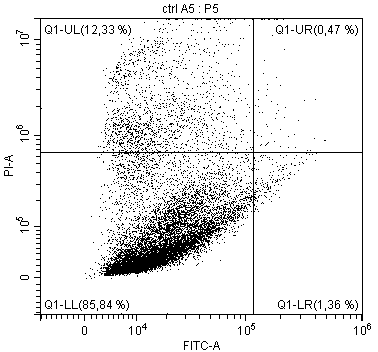  A |  |
| --- | --- |
| 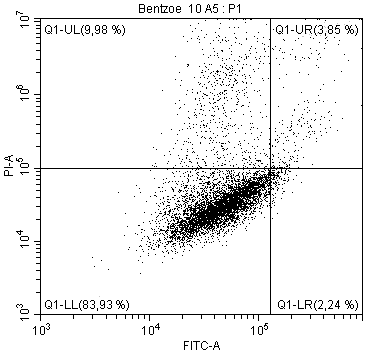  B | 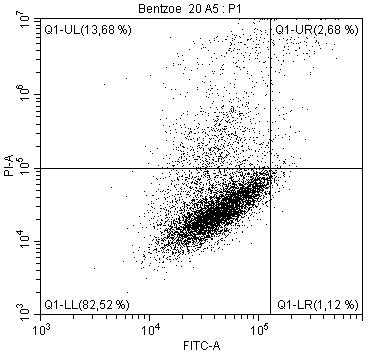  C |
| 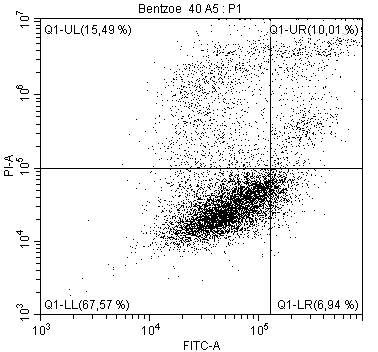  D | 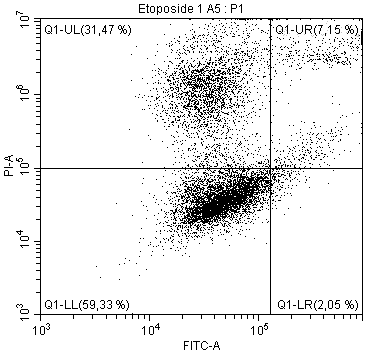  E |
| 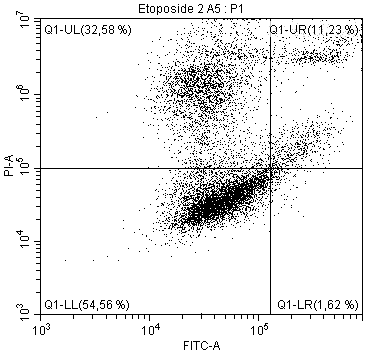  F | 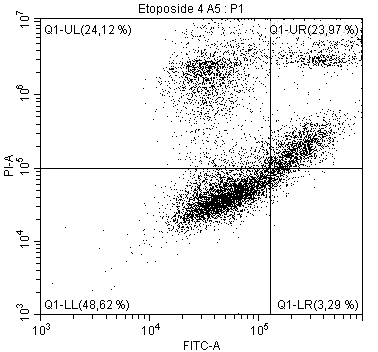  J |

**Figure S2: Representatives annexin V-FITC/PI flow cytometric profile of HepG2 cells, 48 hours post extract/control treatment.** Cells were treated for 48 hours with (A) negative control (0.025% DMSO); (B, C, D) 10 µg/mL, 20 µg/mL and 40 µg/mL of *T. bentzoë*, respectively; (E, F, G) 1 µg/mL, 2 µg/mL and 4 µg/mL of etoposide, respectively.

| 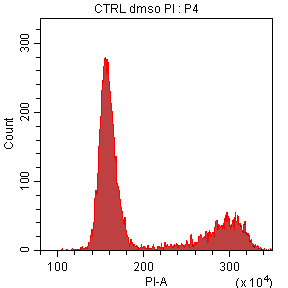  A |  |
| --- | --- |
| 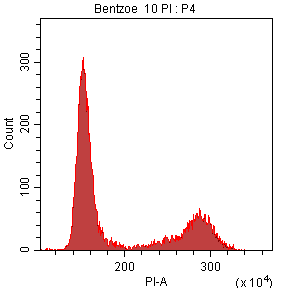  B | ***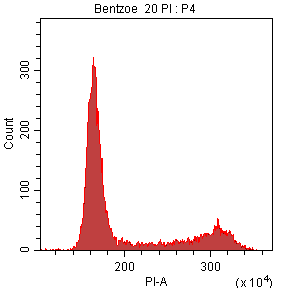***  C |
| 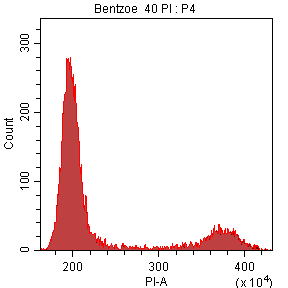  E | 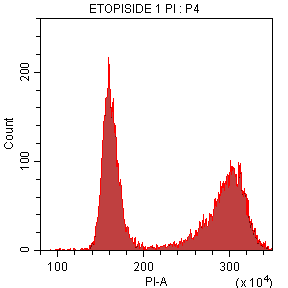 |
| ***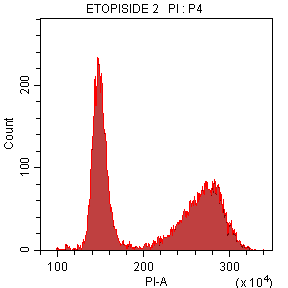***  D  F | 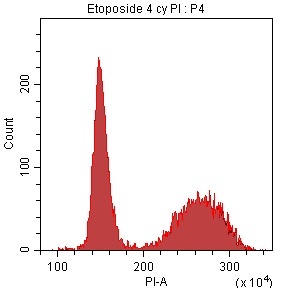  G |

**Figure S3: Representatives cell cycle histogram of HepG2 cells, 48 hours post extract/control treatment.** Cells were treated for 48 hours with (A) negative control (0.025% DMSO); (B, C, D) 10 µg/mL, 20 µg/mL and 40 µg/mL of *T. bentzoë*, respectively; (E, F, G) 1 µg/mL, 2 µg/mL and 4 µg/mL of etoposide, respectively.


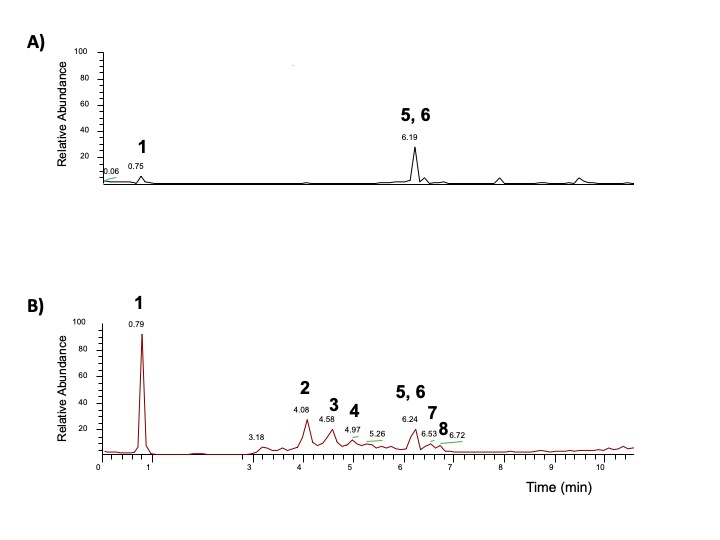


**Figure S4: UPLC ESI MS of the butanol fraction 6 at the positive (A) and negative (B) mode.** The identified compounds are indicated in Table 6.


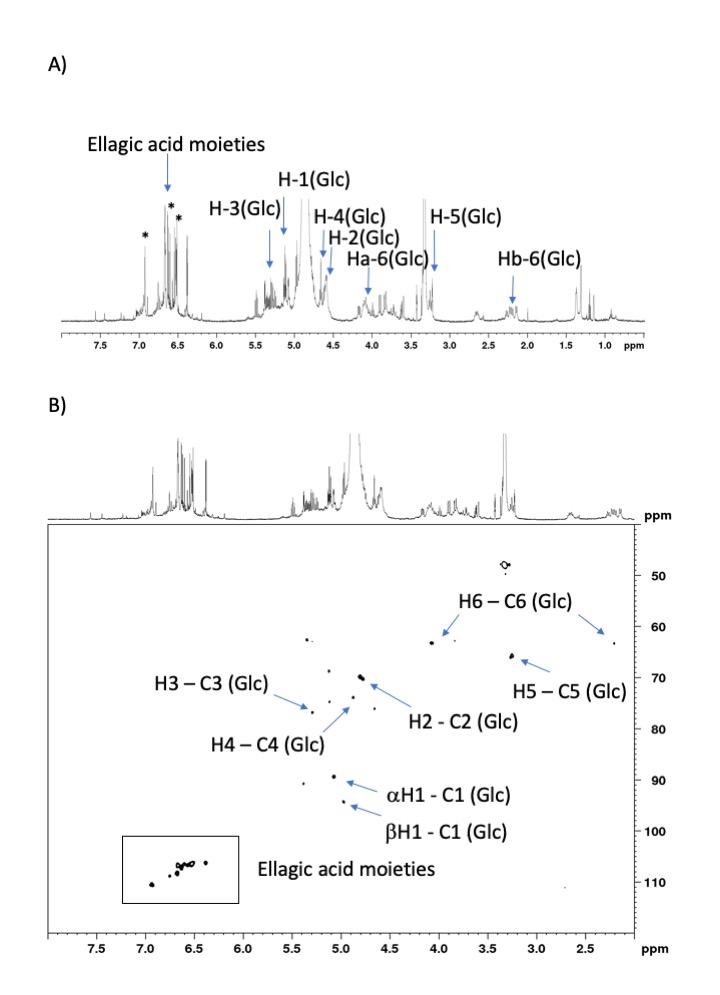


**Figure S5: ^1^H NMR spectrum (A) and HSQC spectrum (B) for α and β-punicalagin (1) present in the HPLC fraction at RT 2-3 min in methanol-d_4_.** The ^1^H NMR peaks and HSQC correlation spots are consistent with those of punicalagin [1–5].


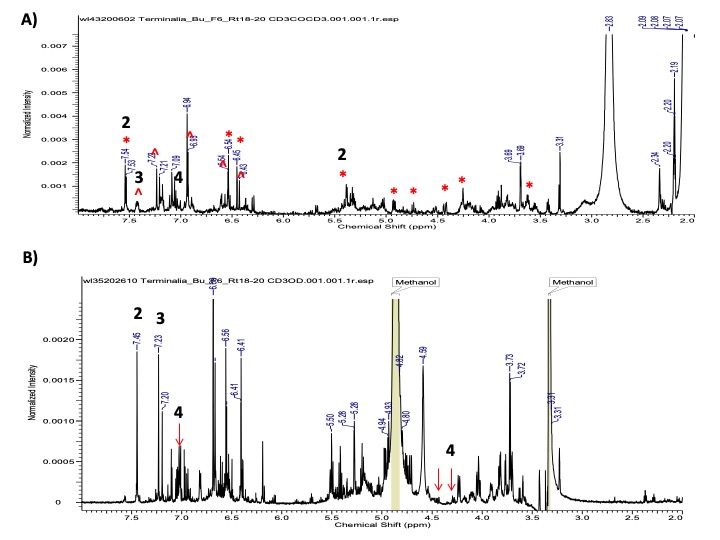


**Figure S6: ^1^H NMR spectra for a mixture of isoterchebulin (2), terflavin A (3), and**

**3,4,6-trigalloyl-beta-D-glucopyranose (4) in a HPLC fraction at RT18-20 min in acetone-d_6_ (A) and methanol-d_4_ (B).**The NMR peaks masked as * and ^ in acetone-d_6_ are consistent with those of isoterchebulin (**2**) [1] and terflavin A (**3**) [6], respectively. The ^1^H NMR peaks measured in methanol-d_4_ (B) are consistent with those of 3,4,6-trigalloyl-beta-D-glucopyranose (**4**) [7].


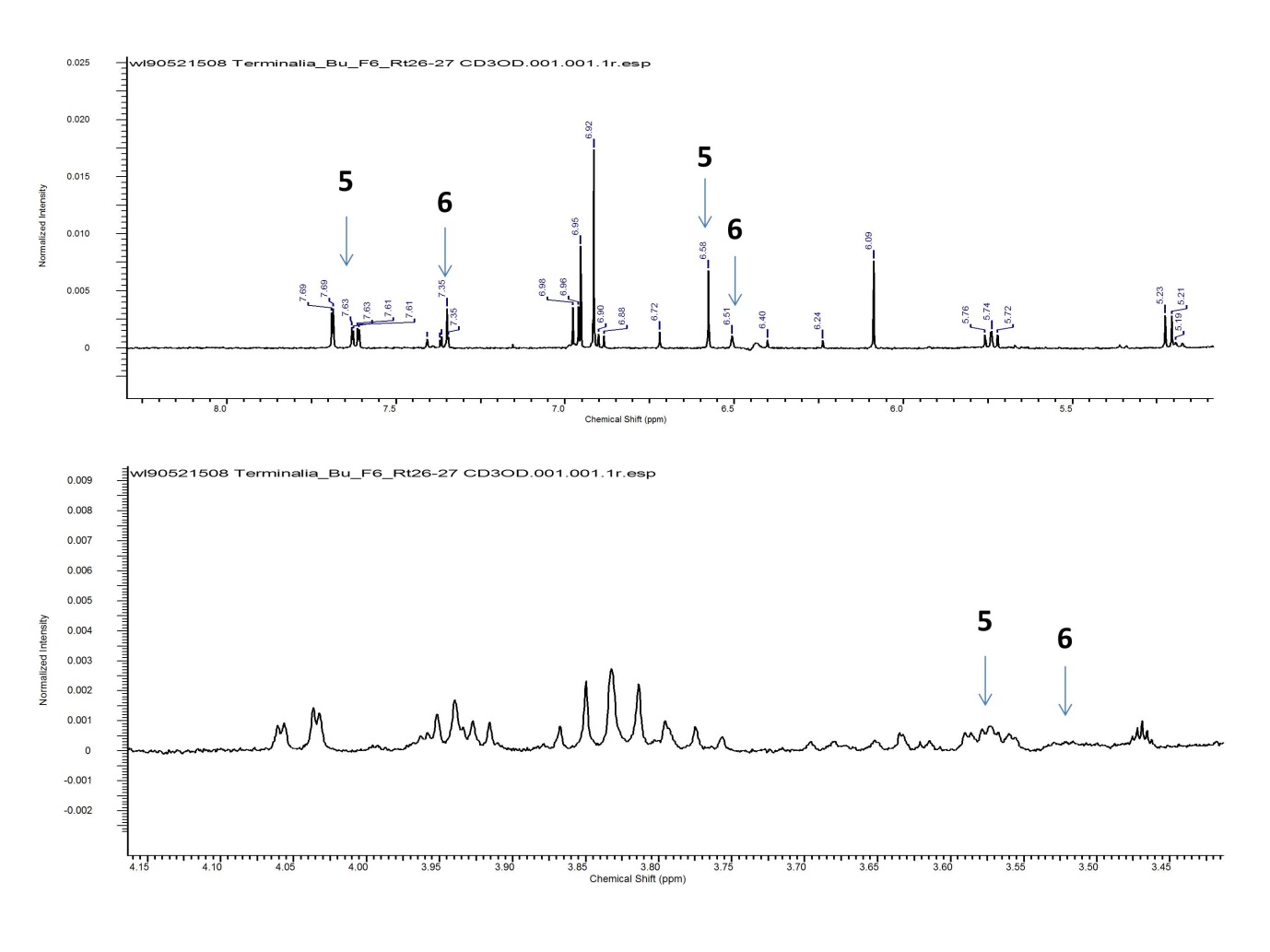


**Figure S7: ^1^H NMR spectra for a mixture of 2"-O-galloyl-orientin (3) and 2"-O-galloyl-isoorientin (6) with a ratio of 5:2 based on the integration of single hydrogen in each compound.** The assignment of each peak is listed in **Table S1**.


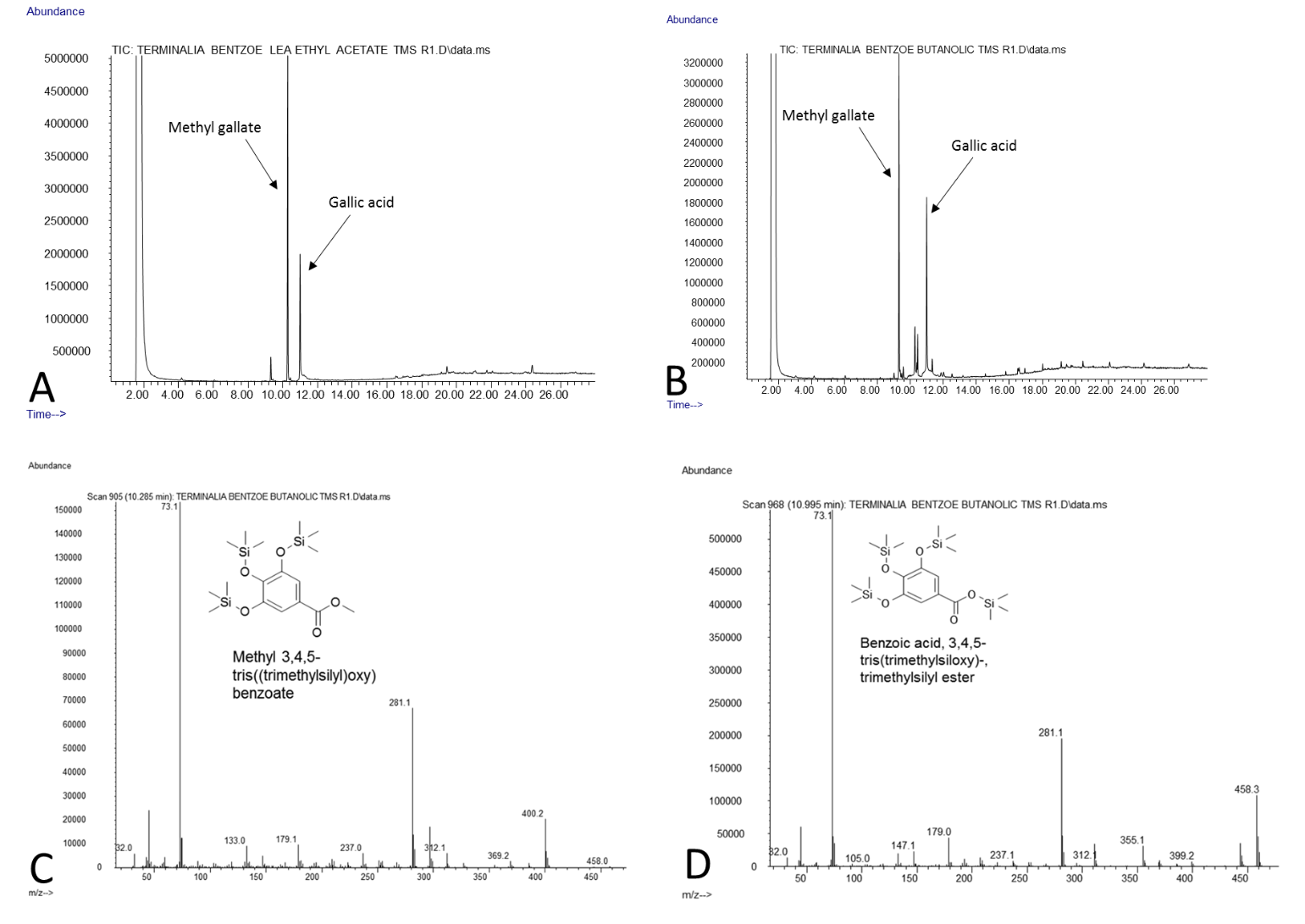


**Figure S8: GC-MS chromatogram of TMSi derivatives of ethyl acetate (A) and butanol (B) fraction of *T. bentzoë*.** Mass spectrum of the peak for TMSi derivative of methyl gallate (C) and gallic acid (D) present in both organic fractions. Methyl gallate, GC-MS of TMSi derivative of methyl gallate, Rt, 11.030 min, *m/z* 400.2 [M]^+^, 369.2, 312.2, 281.1,179.1, 133.0, 73.1. The data are consistent with those stored in the NIST 2011 Mass Spectral Library (Agilent Technologies, USA). Gallic acid, GC-MS of TMSi derivative of gallate, Rt, 10.320 min, *m/z* 458.1 [M]^+^, 355.1, 314.9, 281.1, 73.1. The *m/z* pattern is consistent with those previously reported [8].

**Table S1 ^1^H NMR spectral data (500 MHz, CD_3_OD) of the O-galloyl-C-glycosylflavones 7 and 8 (5:2) [" in ppm, multiplicities and *J* values (Hz) are given in parentheses].**

| proton | 7 | 2”-O-Galloylorientin  [2] | 8 | 2”-O-Galloylisoorientin  [2] |
| --- | --- | --- | --- | --- |
| 3 | 6.58, 6.40^a^ (s) (10:1) | 6.55, 6.37a (s) | 6.51 (s) | 6.45 (s) |
| 6 | 6.09, 6.24^a^ (s) (10:1) | 6.06, 6.21^a^ (s) | 7.35 (d; 2.0) | 7.34 (d; 2.0) |
| 8 |  |  | 6.43 (s) | 6.39 (s) |
| 2’ | 7.69, 7.37^a^ (d; 2.2) | 7.68, 7.38^a^ (d; 1.9) |  |  |
| 5’ | 6.97 (d; 8.5) | 6.97 (d; 8.4) | 6.89 (d; 8.0) | 6.89 (d; 8.9) |
| 6’ | 7.62, 7.40^a^ (dd; 8.4, 2.2) | 7.61, 7.40^a^ (dd; 8.4, 1.9) | 7.36 (dd; 8.0, 2.0) | 7.35 (dd; 8.9, 2.0) |
| *Glucosyl* |  |  |  |  |
| 1’’ | 5.22 (d; 10.1) | 5.22, 5.35^a^ (d; 10.1) | 5.18 (d; 10.1) | 5.16 (d; J=10.0) |
| 2’’ | 5.74 (t; 10.1) | 5.74, 5.66^a^ (t; 9.7) | 5.74 (t; 9.1) | 5.72 (br t) |
| 3” | 3.85 (t; 9.0) | 3.83 (t; 9.0) | 3.78 (t; 9.3) | 3.76 (t; 9.6) |
| 4” | 3.81 (t; 9.0) | 3.79 (t; 9.0) | 3.63 (t; 9.4) | 3.61 (t; 9.4, 9.1) |
| 5” | 3.57 (m) | 3.55 (m) | 3.52 (m) | 3.50 (m) |
| 6a” | 4.05 (dd; 2.2, 12.1) | 4.02 (dd; 1.9, 12.1) | 3.93 (overlapped) | 3.93 (dd; 12.0, 1.8) |
| 6b” | 3.93 (dd; 6.0, 12.1) | 3.91 (dd; 5.8, 12.1) | 3.80 (overlapped) | 3.79 (dd; 5.5, 12.0) |
| *Galloyl* |  |  |  |  |
| 2’’’ | 6.92, 6.72^a^ (s) | 6.89, 6.69^a^ (s) | 6.95 (s) | 6.93 (s) |
| 6” | 6.92, 6.72^a^ (s) (10:1) | 6.89, 6.69^a^ (s) | 6.95 (s) | 6.93 (s) |

1. Apel, C.; Bignon, J.; Garcia-Alvarez, M.C.; Ciccone, S.; Clerc, P.; Grondin, I.; Girard-Valenciennes, E.; Smadja, J.; Lopes, P.; Frédérich, M.; et al. N-myristoyltransferases inhibitory activity of ellagitannins from Terminalia bentzoë (L.) L. f. subsp. bentzoë. *Fitoterapia* **2018**, *131*, 91–95, doi:10.1016/j.fitote.2018.10.014.

2. Latté, K.P.; Ferreira, D.; Venkatraman, M.S.; Kolodziej, H. O-galloyl-C-glycosylflavones from Pelargonium reniforme. *Phytochemistry* **2002**, *59*, 419–424, doi:10.1016/S0031-9422(01)00403-4.

3. Liu, M.; Katerere, D.R.; Gray, A.I.; Seidel, V. Phytochemical and antifungal studies on Terminalia mollis and Terminalia brachystemma. *Fitoterapia* **2009**, *80*, 369–373, doi:10.1016/j.fitote.2009.05.006.

4. Marzouk, M.S.A.; El-Toumy, S.A.A.; Moharram, F.A. Pharmacologically Active Ellagitannins from Terminalia myriocarpa. *Planta Med.* **2002**, *68*, 523–527, doi:10.1055/s-2002-32549.

5. Doig, A.J.; Williams, D.H.; Oelrichs, P.B.; Baczynskyj, L. Isolation and structure elucidation of punicalagin, a toxic hydrolysable tannin, from Terminalia oblongata. *J. Chem. Soc. Perkin Trans. 1* **1990**, 2317, doi:10.1039/p19900002317.

6. Tanaka, T.; NONAKA, G.-I.; NISHIOKA, I. Tannins and related compounds. XLII. Isolation and characterization of four new hydrolyzable tannins, terflavins A and B, tergallagin and tercatain from the leaves of Terminalia catappa L. *Chem. Pharm. Bull. (Tokyo).* **1986**, *34*, 1039–1049, doi:10.1248/cpb.34.1039.

7. Yakubu, O.F.; Adebayo, A.H.; Dokunmu, T.M.; Zhang, Y.-J.; Iweala, E.E.J. Cytotoxic Effects of Compounds Isolated from Ricinodendron heudelotii. *Molecules* **2019**, *24*, 145, doi:10.3390/molecules24010145.

8. Johnson-ajinwo, O.R.; Richardson, A.; Li, W.-W. Cytotoxic effects of stem bark extracts and pure compounds from Margaritaria discoidea on human ovarian cancer cell lines. *Phytomedicine* **2015**, *22*, 1–4, doi:10.1016/j.phymed.2014.09.008.
